# Psychological Capital and Organizational Citizenship Behaviors of Construction Workers: The Mediating Effect of Prosocial Motivation and the Moderating Effect of Corporate Social Responsibility

**DOI:** 10.3390/bs13120981

**Published:** 2023-11-28

**Authors:** Wei Su, Juhee Hahn

**Affiliations:** 1 The Graduate School, Chung-Ang University, Seoul 06974, Republic of Korea; suv4591@gmail.com; 2 Department of Business Management, Chung-Ang University, Seoul 06974, Republic of Korea

**Keywords:** positive psychological capital, prosocial motivation, organizational citizenship behavior, corporate social responsibility

## Abstract

Due to construction industry projects’ large-scale, long-period, and outdoor operation characteristics, employees’ organizational citizenship behavior (OCB) plays an essential role in cost-saving, high-efficiency, and environmentally friendly development strategies. This study discusses how to improve employees’ OCB from two levels of employees’ psychological factors and corporate social responsibility (CSR) at the organizational level. We verified this study’s hypotheses based on 336 valid questionnaires collected from 56 teams. The results indicated that (1) positive psychological capital (PsyCap) was a positive predictor of employees’ OCB; (2) PsyCap was positively related to employees’ prosocial motivation, and prosocial motivation partially mediated the relationship between PsyCap and employees’ OCB; and (3) CSR moderated the relationship between PsyCap and prosocial motivation and played a significant moderating role between prosocial motivation and OCB. These findings provide an empirical research basis for the theories of conservation of resources (COR), self-determination, and affective events. This research also has managerial implications for improving employees’ OCB in the construction industry.

## 1. Introduction

Projects in the construction industry are usually large-scale and long-term. Team members help one another maintain harmonious interpersonal relationships for the smooth completion of a project. Furthermore, the particularity of outdoor work in the construction industry often makes workplaces face force majeure factors such as weather or natural disasters, so companies require employees to take the initiative to protect property on the construction site. Moreover, because construction teams usually have to complete projects with limited material and financial resources, consciously protecting and saving company resources can save construction companies huge costs [1]. Unfortunately, the organization cannot reflect these OCBs of timely response to accidents or active teamwork in its compensation system [2]. However, these behaviors can significantly improve the organizational performance of its construction team. Given the importance of employees’ OCB in the construction industry, this article discusses the leading elements of promoting employees’ OCB from two levels: employees’ characteristics, and organizational values.

In the past, scholars of psychology focused on solving people’s pain and preventing employees from engaging in damaging behavior. However, with the development of positive psychology [3], scholars began emphasizing the need to pay attention to the positive mental state of employees in the workplace and the positive organizational behavior triggered by positive emotions [4]. Psychological capital is “an individual’s positive psychological state of development”, consisting of four dimensions: “Self-efficacy”, “optimism”, “hope”, and “resilience” [5]. Researchers regard positive psychological capital (PsyCap) as an important personal resource, which refers to a person’s ability to find various ways (hope) to achieve success [6]. PsyCap also refers to quickly recovering from setbacks, having the confidence to achieve goals (self-efficacy), not being afraid to try new methods, and remaining optimistic about the future to maintain a positive direction [5]. These qualities are connected and combined to help individuals achieve success [7,8]. According to its original definition [9], OCB is an organization-oriented positive behavior, so it is reasonable to regard PsyCap as the leading factor of OCB. This study draws on the conservation of resources (COR) theory to explain the relationship between PsyCap and OCB. That is, people with rich personal resources are more willing to invest resources in the organization to obtain more resources.

In the relationship between PsyCap and OCB, researchers tested the roles of organizational identity [10], work engagement [11], organizational trust [8], perceived organizational support [12], and authentic leadership [13]. Although there have been many studies on the relationship between PsyCap and OCB [11], previous research [14] found that the relationship between prosocial motivation and OCB was mediated by other variables and stated that OCB in relation to the effect of prosocial motivation remains scarce. Understanding the potential mechanism linkages between PsyCap and trait-like counterparts (prosocial motivation in this study) may increase the utility of PsyCap in terms of enhancing the links between personal traits (prosocial motivation) and desirable outcomes (OCB) [15]. Prosocial motivation refers to the desire to care about the wellbeing of others, which is a positive attitude of the individual [16]. The affective events theory emphasizes that the emotional conditions experienced by employees in the workplace play a key role in their behaviors and attitudes [17]. This study assumes that employees with a high level of PsyCap can maintain more positive psychological conditions in the workplace and, therefore, have more positive attitudes towards the people and things around them. Therefore, this study proposes that PsyCap can promote the generation of prosocial motivation to improve employees’ OCB.

Construction industry activities can cause many problems, such as destroying biodiversity, emitting greenhouse gases, producing industrial waste [18,19], and causing health and safety accidents [20]. Therefore, due to the huge impact of construction activities on society and the environment, corporate social responsibility (CSR) in the construction industry has received widespread attention from academia and industry [21]. The existing literature mainly emphasizes the effects of CSR activities on the company’s profitability and the behavior of external stakeholders [22]. However, very few studies have investigated the influence of CSR activities on the company’s internal stakeholders, such as employees’ behavior, attitudes, and commitment [23]. When an organization fulfills its social responsibilities, employees will view management who fulfill CSR as role models and identify with their organizations that demonstrate a positive prosocial value and reputation [24]. Employees with prosocial motivation establish a high degree of identity in organizations that value CSR, and they will be more engaged in extra actions [25]. Consequently, we propose that they will actively respond to CSR activities through prosocial action (OCB) in the organization. Therefore, this study expands the existing literature by focusing on the internal stakeholders of CSR (employees) and uses social learning theory, social identity, and person–organization (PO) fit to explore the influence of CSR on employees’ prosocial motivation and OCB.

Based on the above, this study focuses on employees in the Chinese construction industry to explore the following questions:

**Q1.**  *What role does positive psychological capital play in promoting employees’ OCB?*

**Q2.**  *Does individual prosocial motivation explain the influence of positive psychological capital on employees’ OCB?*

**Q3.**  Can *CSR, as an organizational-level prosocial variable, amplify or weaken the links between positive psychological capital, prosocial motivation, and OCB?*

## 2. Literature Background and Hypothetical Development

### 2.1. Employees’ Positive Psychological Capital and Employees’ OCB

“Self-efficacy”, “optimism”, “hope”, and “resilience” are the four dimensions of PsyCap. According to Luthans et al. [5], these dimensions, representing “a state of positive psychological development”, are characterized by (1) self-efficacy: confidence in taking effective measures to deal with challenging tasks successfully; (2) optimism: a positive attribution for succeeding now and in the future; (3) hope: some persisting life goals, keep moving towards these goals to achieve success; and (4) resilience: when encountering obstacles and difficulties, the ability to sustain and bounce back and even beyond to attain success. Thus, these dimensions play a significant role in promoting positive outcomes [26].

Organ [27] defined OCB as “contributions to the maintenance and enhancement of the social and psychological context that supports task performance”. This refers to the voluntary, discretionary, and altruistic activities conducted by employees outside of their job requirements, and for which they may not receive payment or reward [27,28]. The COR theory [29] is particularly valuable for understanding how PsyCap affects OCB. OCB is a behavior that arises due to abundant resources [13]. Employees with high levels of PsyCap (self-efficacy, hope, optimism, and resilience) are full of hope even under adverse circumstances and try to focus on the positive aspects when they find themselves working in unfavorable environments and conditions. They are optimistic about the future, believing that they can overcome situations and recover from negative emotional experiences [30]. Thus, PsyCap can help people preserve and develop their resources, motivating them to demonstrate OCB to increase other work resources (e.g., good interpersonal relationships) and satisfy basic psychological needs (e.g., relatedness) [31]. Participating in OCB has been proven to require many human and financial resources and to make employees exhausted [32]. Therefore, high-level PsyCap is a resource to help employees practice OCB more [33].

Newman, Ucbasaran, Zhu, and Hirst [34] found that employees with higher levels of PsyCap have a more positive outlook on people and events, and that they participate in more helpful behavior toward their coworkers. PsyCap helps employees extend their efforts to support their colleagues in achieving their goals. This will strengthen the interpersonal relationships between colleagues and encourage employees to thrive [32]. Occupational psychologists believe that high-PsyCap employees demonstrate more OCB because they can put more physical and cognitive energy into their work [35]. According to Karatepe and Karadas [36], employees with solid PsyCap are more satisfied with their jobs and engage in high-level extra-role behaviors:

**Hypothesis** **1.** *Employees’ PsyCap is positively related to employees’ OCB.*

### 2.2. Mediation of Prosocial Motivation in the Relationship between Employees’ PsyCap and Employees’ OCB

Prosocial motivation is “the desire to protect and improve the well-being of other people” [16]. As the definition says, prosocial motivation is a psychological state in which people benefit others because they care about others’ wellbeing [37]. There are three parts to prosocial motivation: (1) global prosocial motivation: employees’ intention to contribute to the organization, e.g., to work hard for the development of the company; (2) contextual prosocial motivation: employees’ intention to benefit a specific group of people, e.g., to help all of their subordinates to complete tasks; and (3) situational prosocial motivation: employees’ intention to benefit a specific individual, e.g., to only help one subordinate complete their task [16].

According to motivation theory, motivation is the basic drive for our behaviors [38]. We believe that PsyCap also has an influence on OCB through prosocial motivation. Prior research found that individuals with high levels of self-efficacy showed more prosocial tendencies, such as “cooperating and sharing, being helpful, and caring about the interests of others” [39,40]. Hopeful employees are psychologically strong enough to help others and will be motivated by their beliefs. Optimism can improve employees’ self-esteem and morale, creating a positive psychological experience [7]. Resilience helps people recover quickly from adversity, which ultimately leads to increased responsibility. This increased level of responsibility promotes prosocial motivation [41]. Nawaz et al. [30] found that employees with greater levels of PsyCap interpret people and events more positively. Thus, PsyCap can reinforce the beneficial link between prosocial motivation and the ability to thrive at work. High PsyCap is a precious personal resource that allows people to maintain a positive attitude. Therefore, we believe that such a positive state of mind will promote prosocial motivation.

According to self-determination theory, objectives with more autonomous reasons are more likely to translate into behaviors. Therefore, autonomic motivation for prosocial behavior (i.e., prosocial motivation) can be predicted to promote more prosocial behavior [42]. Rioux and Penner [43] proposed that social values (e.g., the desire to help others) are key motivations for demonstrating OCB. People driven by highly prosocial motives are more likely to consider the needs of others, perceive their surroundings in a caring way, and then perform helpful behaviors [44]. An individual with a high prosocial motivation tends to impact others positively, motivating employees to participate in OCB more frequently [45,46]. Those who are less prosocially motivated, on the other hand, may be more concerned with their ambitions and less concerned with what others think or need. Previous research has shown that employees with higher prosocial motivations will help their colleagues [43], and that those with prosocial motivations pay more attention to the preferences of their colleagues [47]. Helping colleagues is an important part of good OCB. Arshad, Abid, and Torres [14] indicated that prosocial motivation promotes OCB through ethical leadership and leader–member exchange. Employees with scarce prosocial motivations were less likely to establish OCB. According to the above, we propose the following hypotheses:

**Hypothesis** **2.** *Employees’ PsyCap is positively related to employees’ prosocial motivation.*

**Hypothesis** **3.** *Employees’ prosocial motivation mediates the relationship between employees’ PsyCap and employees’ OCB.*

### 2.3. Moderation of CSR

CSR refers to all prosocial organizational activities and outcomes not pursuing profit maximization [48]. Werther and Chandler [49] defined CSR as internal CSR and external CSR based on stakeholder perspectives. External CSR focuses on the local community (e.g., charitable donations, community development investment), the environment (e.g., environmental protection investment, pollution prevention), and consumers (i.e., responsibility to consumers of company products or services) [50]. Internal CSR focuses on internal resources (i.e., employees). Most of the previous studies focused on external CSR, paying attention to financial performance and marketing effects. However, employees are also essential stakeholders of CSR because they are influenced by and impact their employer’s CSR actions [51]. Recent studies have begun to examine the psychology of CSR, focusing on how employees perceive and respond to CSR [52]. When an organization fulfills its social responsibilities, such as protecting the environment, actively participating in donation activities, taking care of people in need, etc., employees will feel that the organization is responsible and trustworthy, so they will feel psychologically safer to allocate their resources and energy to the organization. Therefore, we believe that employees with positive psychological capital will be more willing to show prosocial motivations when organizations perform CSR.

According to social learning theory [53], people primarily learn how to behave in social situations through the influences of examples or models. Therefore, in an organization that values and emphasizes CSR and regards CSR as a standard of conduct, subordinates will view leaders and management who fulfill CSR as role models [54]. In addition, employees will have a greater understanding of their expected appropriate and normative behaviors in the organization and, thus, will be more likely to have a prosocial motivation to care about the welfare of others [23]. In addition to social learning theory, social identity theory can also illustrate the relationship between CSR and prosocial motivation. For example, social identity theory [55] points out that employees usually identify with and commit to organizations that demonstrate positive organizational values and reputation [56], so as to establish or enhance a positive self-concept. Moreover, when an organization fulfills its sense of social responsibility, its positive evaluation will increase employees’ recognition of the company’s ethical practices [22], thereby increasing their prosocial motivation.

**Hypothesis** **4.** *CSR moderates the relationship between employees’ PsyCap and employees’ prosocial motivation. Positive relationships between employees’ PsyCap and employees’ prosocial motivation will be stronger when CSR is high rather than low.*

A person–organization (PO) fit [57] refers to the compatibility between people and organizations. This definition includes mutual needs satisfaction, value consistency between individuals and organizations, personality similarities between individuals and other organization members, and shared goals between individuals and organizations. The concept of CSR at the organizational level is consistent with the prosocial motivation at the individual level. That is, prosocial motivation is the prosocial orientation of the individual, while CSR focuses on the prosocial orientation of the organization [58,59]. When employees realize that their prosocial motives are consistent with the organization’s prosocial motives, this consistent belief will make it easier for employees and the organization to build mutual trust, reduce conflicts in the work process, and improve the quality and efficiency of interactions [60,61]. Therefore, we believe that this positive interaction will encourage employees to contribute to the organization.

Shao et al. [52] found that when employees perceive CSR as high rather than low, the positive relationship between employees’ prosocial motivation and organizational commitment will be stronger. Employees with prosocial motivation establish a high degree of identity in organizations that value CSR, and they will be more engaged in off-role actions [23,62]. Kim and Kim [63] indicated that employees with highly prosocial motivations may want to contribute to others, so they may think that the organizations’ ethical practices are closely related to the values that they pursue. Then, they will actively respond to CSR activities through prosocial action (OCB) in the organization.

**Hypothesis** **5.** *CSR moderates the relationship between employees’ prosocial motivation and their OCB. Positive relationships between employees’ prosocial motivation and OCB will be stronger when CSR is high rather than low.*

**Figure 1:**
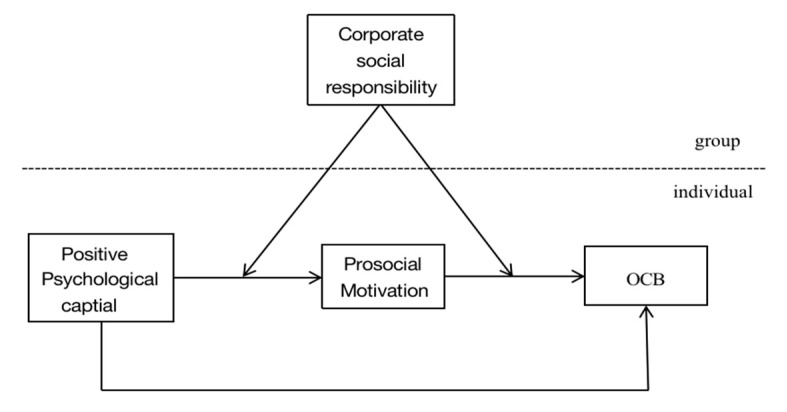
Research model.

Figure 1 shows the research model.

## 3. Methods

### 3.1. Sample and Procedure

We collected the study’s data from fifteen large Chinese state-owned construction enterprises located in Beijing, Shanghai, and Guangdong Province, China, in 2023. First, we contacted the human resources department of each company. Subsequently, after the team manager’s approval, the human resources department provided us with the construction project manager’s contact information. Data were collected from two sources (team leader and team member) in three waves (two weeks apart) to minimize common-method bias, as recommended by Podsakoff et al. [64]. Team leaders were invited to rate the CSR, and team members were invited to evaluate positive psychological capital at the first point in time (from 20 February to 3 March). Two weeks later, we ran the second survey to invite team members to rate their prosocial motivation (from 20 March to 31 March), and the third survey after 2 weeks invited team leaders to evaluate subordinates’ OCB (from 17 April to 28 April).

At the beginning of the questionnaire, we explained the purpose of this research and ensured the anonymity and confidentiality of all participants. After the participants read and agreed to our statement, they completed the questionnaire. When we sent the questionnaires to the team leaders, we provided them with the different team numbers. We asked the team leaders to inform their team members of their respective team numbers, allowing respondents to answer the question “Please write down your team number” in the questionnaire. Therefore, we could use the team numbers to match the data of team leaders and team members. We obtained 378 responses from 63 teams. After removing invalid and missing questionnaires, we had 336 valid questionnaires from 56 teams.

We used SPSS 26.0 for descriptive statistical analysis. Among the 56 team leaders, 83.9% (N = 47) were male and 16.1% (N = 9) were female. The team leaders’ ages mainly ranged from 31 to 40 years old (78.5%, N = 44), followed by the 41–50-year range (16.1%, N = 9) and the 21–30-year range (5.4%, N = 3). Regarding the team leaders’ educational level, 16.4% (N = 10) had college degrees, 59.0% (N = 36) had bachelor’s degrees, and 16.4% (N = 10) had master’s degrees. Among the 336 team members, 75.3% (N = 253) were male and 24.7% (N = 83) were female. The team members’ ages included 34.8% (N = 117) in the 21–30-year range, 63.4% (N = 213) aged 31–40 years, and 1.8% (N = 6) aged 41–50 years. Regarding the team members’ work experience, 41.7% (N = 140) had work experience of 1–5 years, 30.9% (N = 104) had 6–10 years, and 27.4% (N = 92) had 11–15 years. Finally, the team members’ education levels included 10.7% (N = 36) high school graduates or below, 30.4% (N = 102) with college degrees, 57.1% (N = 192) with bachelor’s degrees, and 1.8% (N = 6) with master’s degrees.

### 3.2. Measures

This study used a 24-item scale in four dimensions (self-efficacy, hope, resilience, and optimism), developed by Luthans et al. [65], to measure employees’ PsyCap. A sample item of self-efficacy is “I feel confident helping to set targets/goals in my work area”. A sample item of hope is “I now work enthusiastically to achieve my work goals”. A sample item of resilience is “I was able to recover from a setback at work and move on”. A sample item of optimism is “I expect the best possible result in the situation that my task results are uncertain”. We assessed prosocial motivation using Grant’s [66] 5-item scale. Sample items included “I will do my best to do work that is beneficial to others”. We assessed OCB on an 11-item scale developed by Lee and Allen [67]. A sample item is “This employee willingly gives his/her time to help others who have work-related problems”. We adopted a 17-item scale in four dimensions (CSRS, CSRE, CSRC, and CSRG), developed by Turker [68], to measure CSR. Since construction companies generally operate in a project-based mode, each project is regarded as a separate entity with its own goals, spending limit, schedule, and resource needs. Due to the characteristics of outdoor work in the construction industry, each project team goes to a certain place to work independently. Employees in the same construction company will belong to different project teams. Therefore, what employees can observe and contact most in their daily work is whether the project team that they belong to has fulfilled their social responsibility obligations. Therefore, we adjusted the CSR of the entire organization to a team CSR centered on the project group. CSRS means CSR to society; a sample item is “Our team contributes to campaigns and projects that promote the well-being of the society”. CSRE means CSR to employees; a sample item is “Our team is primarily concerned with employees’ needs and wants”. CSEC means CSR toward customers, and a sample item is “Customer satisfaction is highly important for our team”. Finally, CSRG means CSR toward the government, and a sample item is “Our team always pays taxes on a regular and continuing basis”. The participants used a five-point Likert scale (“1” = strongly disagree, “5” = strongly agree) to respond to these statements.

### 3.3. Analysis Strategy

A confirmatory factor analysis was performed using MPlus 8.3 to evaluate the validity of the model fit indices. We used the following goodness-of fit-statistics criteria [69] to assess the model fitness: chi-squared goodness of fit/degrees of freedom (χ^2^/df) must be <3, the Tucker–Lewis fit index (TLI) and comparative fit index (CFI) must be above 0.9, and the standardized root-mean-square residual (SRMR) and the root-mean-square error of approximation (RMSEA) must be <0.08 [69]. The results showed that χ^2^/DF = 1.080 (<3), CFI = 0.994 (>0.9), TLI = 0.992 (>0.9), RMSEA = 0.016 (<0.08), and SRMR = 0.032 (<0.08).

We also used composite reliability (CR), average variance extracted (AVE), and Cronbach’s alpha to confirm the constructs’ validity and reliability. In addition, we undertook a multilevel path analysis to test the hypotheses in MPLUS8.3. Harman’s [70] one-factor test was used to check the common-method variance in this study. The unrotated factor solution indicated that one factor explained 29.74% of the variance, significantly below the 50% threshold, revealing that common-method variance was not a problem in this study.

## 4. Results

### 4.1. Preliminary Analyses

As shown in Table 1, we first tested the questionnaires’ reliability and validity. All of the Cronbach’s alpha values exceed 0.70 [71]. Therefore, the internal consistency of all of the variables was confirmed. Furthermore, all of the average variance extracted (AVE) values were above 0.50, and all CR values were above 0.70 [72]; thus, all of the constructs’ reliability and validity scores are acceptable.

As shown in Table 2, the standard deviations of all of the variables were within the normal range, and the variables showed the expected binary correlation. In addition, the square roots of the AVE values displayed on the diagonal line exceeded the values of the correlations, proving the discriminant validity [73]. Thus, the data were suitable for further analysis.

### 4.2. Hypothesis Tests

The study followed the well-known methodology of Baron and Kenny [74] to test the mediating effect of prosocial motivation, conducting four regressions as follows: Path c: the total impact of the independent variable on the dependent variable; Path a: the influence of the independent variable on the mediating variable; Path b: the influence of the mediating variable on the dependent variable; and Path c’: the direct impact of the independent variable on the dependent variable, controlling Paths a and b.

The verification result of the mediation effect is shown in Table 3. The regression coefficient of PsyCap and OCB is 0.248 (*p* < 0.001); thus, a 1-point increase in PsyCap is associated with a 0.248-point increase in OCB. This finding supports H1. The PsyCap and prosocial motivation regression coefficient is 0.174 (*p* < 0.01); a 1-point increase in PsyCap means a 0.174-point increase in prosocial motivation. This finding supports H2. In H3, we assumed that prosocial motivation mediates the relationship between PsyCap and OCB. Our results showed a statistically significant positive mediation effect (PsyCap→PM→OCB) of 0.07 (*p* < 0.01). After adding prosocial motivation, the effect of PsyCap on OCB reduced from 0.248 to 0.178, still reaching a significant level at 0.01. This finding indicated that prosocial motivation was a partial mediating variable, and the partial mediating effect was 28.2% (0.07/0.248 = 0.282). Thus, this finding supports H3.

**Figure 2:**
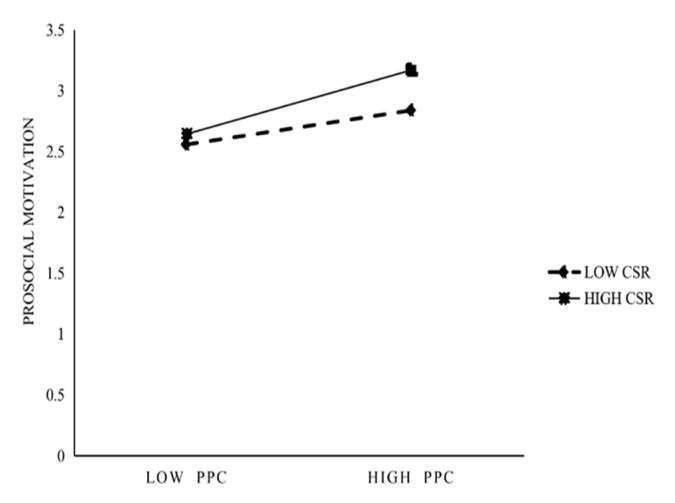
Moderation of CSR in positive psychological capital on prosocial motivation.

**Figure 3:**
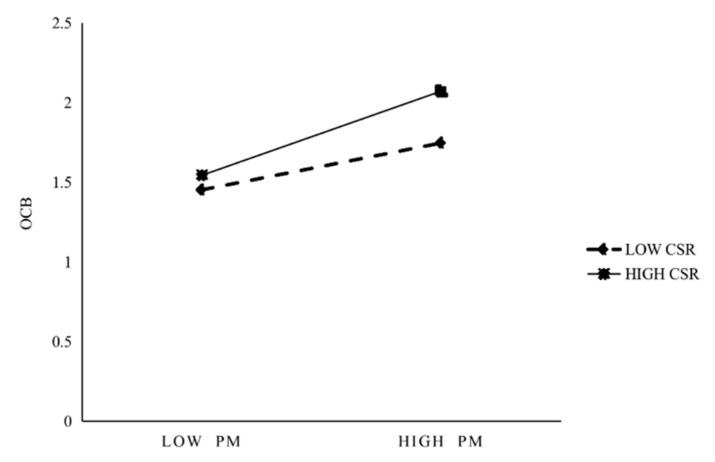
Moderation of CSR in prosocial motivation on OCB.

H4 and H5 state that CSR moderates the effect of PsyCap on prosocial motivation and the effect of prosocial motivation on OCB, respectively. This study first created the interaction term between PsyCap and CSR and found their interaction term to be significantly (r = 0.143, *p* < 0.05) related to prosocial motivation (see Table 4). As shown in Figure 2, compared with low CSR, when CSR is high, the increase in PsyCap will cause more prosocial motivation. Thus, this finding supports H4. The interaction term of prosocial motivation and CSR is significantly (r = 0.157, *p* < 0.01) related to OCB (see Table 4). As shown in Figure 3, compared with low CSR, when CSR is high, the increase in prosocial motivation will cause more OCB. Thus, H5 is supported.

## 5. Discussion

The present study examined the multilevel mechanism of the relationship between PsyCap and OCB, mediated by prosocial motivation and moderated by CSR. Based on the empirical analysis, we can summarize the main results of this study as follows: First, employees’ PsyCap has a significant positive effect on prosocial motivation and OCB. Second, employees’ prosocial motivation partially mediates the relationship between their PsyCap and OCB. Third, CSR has a positive moderating impact on the relationship between their PsyCap and their prosocial motivation. When CSR is high, the increase in PsyCap causes more prosocial motivation. Moreover, CSR plays a significant moderating role in the relationship between employees’ prosocial motivation and OCB. When CSR is high, the increase in prosocial motivation causes more OCB.

### 5.1. Theoretical Implications

First, this study verified that PsyCap is an effective and positive predictor of employees’ OCB, consistent with the findings of Gupta, Shaheen, and Reddy [11], Azim and Dora [12], and Ramalu and Janadari [13]. Although previous studies have found a relationship between PsyCap and OCB, only a few studies have focused on OCB in the construction industry. OCB, the dependent variable in our research, plays a crucial role in the construction industry. Therefore, this research adds to the study of OCB in the construction industry by linking positive psychological capital with organization-oriented prosocial behaviors (OCB). In addition, the relationship between PsyCap and OCB verified by this study once again provides an empirical research basis for COR theory. When employees have higher PsyCap, they have more positive psychological resources, encouraging them to invest or return resources to the organization and colleagues through OCB, and helping them obtain more resources (e.g., harmonious interpersonal relationships).

Second, this study found that prosocial motivation is an effective mediating variable between PsyCap and OCB. The results showed that prosocial motivation is positively related to OCB, consistent with the findings of Arshad et al. [75], who indicated that employees with prosocial motivation engage in discretionary behavior among coworkers. To the best of our knowledge, no studies have explored the influence of PsyCap on prosocial motivation. This study fills that gap and is the first attempt to test the effect of prosocial motivation on the relationship between PsyCap and OCB. This conclusion also provides empirical evidence for the affective events theory, which emphasizes that the emotional conditions experienced by employees in the workplace play a key role in their behaviors and attitudes [17]. Employees with high PsyCap can feel more positive emotions in the workplace and maintain more positive attitudes towards the people and things around them. Therefore, it can improve prosocial motivation, which, in turn, promotes employees’ extra-role behaviors. Furthermore, based on self-determination theory, objectives with more autonomous reasons are more likely to translate into behaviors [42]. The results of this study show that autonomous prosocial motivations will translate into more prosocial behaviors, thus providing empirical evidence for self-determination theory.

Third, given that the relationship between employees’ attitudes and behaviors is not solely affected by the individual level, this study considered the moderating role of prosocial values (i.e., CSR) at the organizational level. Previous studies on CSR focused on external stakeholders (macro-level) [22]. This research mainly focused on the attitudes and behaviors of internal stakeholders, which is the micro-level of CSR. Our research is the first to examine the interactive effects of CSR and PsyCap on prosocial motivation, and it enriches the existing literature on CSR. We found that if the organization actively participates in CSR, employees will have increased levels of prosocial motivation. This finding provides support for social learning and social identity theories. People will imitate and learn how to show appropriate behaviors in the organization. Therefore, when the organization exhibits an organizational prosocial attitude (i.e., CSR), employees will imitate and learn this prosocial tendency, generating more prosocial motivations. Moreover, when employees realize that their organization is performing CSR, they will feel that their work is more meaningful. They will be proud of being in such an organization, thereby generating a greater sense of identity with the organization and, thus, arousing more social motivation.

Moreover, in this study, the moderating effect of CSR between prosocial motivation and OCB was effective. Shao et al. [52] indicated that whether or not perceived CSR is beneficial to organizational commitment depends on employees’ characteristics, such as their prosocial motivation. Thus, in assessing the impact of CSR on employees’ behavior, the level of employees’ prosocial motivation should be regarded as a key factor. Employees with prosocial motives can feel the fit of their own values and organizational values in an organization that fulfills CSR. This finding provides empirical evidence for the PO fit theory—that is, the consistent belief between employee and organization will improve the positive interaction, which encourages employees to contribute to the organization.

### 5.2. Practical Implications

Our research showed that PsyCap promotes subordinates’ positive attitudes (prosocial motivation) and positive work behavior (OCB). In view of the difficult outdoor working environment of the construction industry, how to improve the PsyCap of employees is the top priority of the construction industry management. Leaders should pay attention to the psychological state of their employees. Organizations can design and implement employee training and guidance programs to encourage employees to establish and maintain a high level of PsyCap and introduce these programs into the comprehensive employee development plan [15,30]. Employees can learn how to improve self-efficacy, maintain an optimistic attitude, quickly recover from setbacks, and maintain a good level of psychological capital through these programs.

A new generation of employees expects their employers to demonstrate more prosocial behavior and take on more prosocial responsibilities [76]. Given that CSR can actively promote the prosocial motivation of employees, construction companies can fulfill external social responsibilities by making CSR an organizational strategy. For example, by sourcing materials that have been manufactured using less environmentally harmful materials, the overall carbon footprint of a construction project can be decreased. Moreover, waste production and the consumption of natural resources are commonplace in construction projects. Designing out potential waste from the planning stage can benefit the environment and construction companies by reducing unnecessary resource usage. Furthermore, by integrating energy-efficient materials and technologies into construction projects, companies can provide their customers with environmentally friendly and cost-effective buildings. Similarly, by providing workers with training and development opportunities on how to use green building materials and environmentally friendly technologies, as well as promoting the wide application of green building technology in work [77], the organization can provide a timely understanding of subordinates’ needs, provide professional assistance to employees, and create an excellent organizational atmosphere to actively fulfill their internal social responsibility [78]. Employees’ feelings of self-esteem and organizational identity will increase as a result of the organization’s desirable internal and external social duties.

In addition, construction companies should publicize their CSR efforts to their employees and increase employees’ awareness of their prosocial culture. Since employees at construction companies, especially those at the ground level, are probably unaware of the company’s CSR activities, the organization can inform employees about how it contributes to the community and how external stakeholders benefit from these activities [64,79]. Organizations can promote the prosocial motivation of employees by involving them in charitable activities and guiding and educating them on the prosocial aspects of their work

### 5.3. Limitations and Future Directions

Although this research was based on classical theories and provided empirical test results for the hypotheses, it has some limitations. First, although this study used multisource data with different raters, employees rated the independent and mediating variables, while the leaders rated the dependent and moderating variables. We encourage future research to perform other ratings through colleagues. Second, this study mainly focuses on the antecedents of OCB. Scholars have proposed that the outcomes of OCB, especially the negative consequences, are receiving increased attention [80]. Therefore, future research could expand this model by studying the consequences of OCB. In addition, with the emphasis on ESG management, OCBE is receiving more attention. OCBE is employees’ voluntary behavior beyond the job requirements that leads to a favorable environment. Future research could consider using OCBE as an outcome variable to study the validity of the antecedents in this model. Third, although we examined the impact of PsyCap on prosocial behavior within the organization, future research could extend the outcome variables to prosocial behavior outside the organization, such as actively participating in social responsibility activities carried out by the organization to benefit society. Finally, we conducted our study in only one industry in one Asian country. Since China has a high level of collective culture, results may differ depending on a country’s cultural context. PsyCap may be predicted to have a greater influence in more individualistic countries, where cultural values prioritize individual rights above collective responsibilities. In such cultures, certain aspects of self-belief, such as self-efficacy and optimism, will embody these values, in turn motivating the individual to take positive action [34].

## Figures and Tables

**Table 1:** Scales’ reliability and validity.

Variable	Items	Alpha	Factor Loading	CR	AVE
Positive psychological capital	24	0.962	0.745–0.805	0.957	0.599
Prosocial motivation	5	0.905	0.755–0.994	0.911	0.675
OCB	11	0.915	0.650–0.828	0.919	0.508
CSR	17	0.960	0.697–0.847	0.951	0.565

Alpha = Cronbach’s alpha, AVE = average variance extracted, CR = composite reliability, CSR = corporate social responsibility, OCB = organizational citizenship behavior.

**Table 2:** Correlation matrix of this study’s variables.

Variable	Mean	SD	1	2	3	4
Positive psychological capital	3.704	0.781	(0.774)			
Prosocial motivation	3.492	0.819	0.166 **	(0.822)		
OCB	3.686	0.723	0.268 **	0.490 **	(0.713)	
CSR	3.484	0.764	0.005	0.180 **	0.144 **	(0.752)

CSR = corporate social responsibility; OCB = organizational citizenship behavior; square root of AVE presented along the diagonal; ** *p* < 0.01.

**Table 3:** Mediation of prosocial motivation.

	Estimates	95% CI	Remarks
PsyCap → OCB (total effect, c)	0.248 ***	(0.143, 0.348)	Supported (H1)
PsyCap → PM (a)	0.174 **	(0.069, 0.286)	Supported (H2)
PM → OCB (b)	0.405 ***	(0.321, 0.495)	
PsyCap → PM → OCB (a × b)	0.07 **	(0.028, 0.121)	Supported (H3)
PsyCap → OCB (direct effect, c’)	0.178 **	(0.074, 0.282)	

Bootstrap sample = 1000. CI = confidence interval, PsyCap = positive psychological capital, PM = prosocial motivation, OCB = organizational citizenship behavior; ** *p* < 0.01, *** *p* < 0.001.

**Table 4:** Moderation effect of CSR.

	Estimates	95%CI	Remarks
Group × individual → individual			
PsyCap × CSR→ PM	0.143 *	(0.026, 0.313)	Supported (H4)
Group × individual → individual			
PM × CSR → OCB	0.157 **	(0.091, 0.285)	Supported (H5)

Bootstrap sample = 1000. CI = confidence interval, PsyCap = positive psychological capital, PM = prosocial motivation; * *p* < 0.05, ** *p* < 0.01.

## Data Availability

Data will be made available upon request.
